# Effects of Mechanism of Injury and Time from Injury on Functional Outcomes in Traumatic Brain Injury Patients During Long-Term Acute Care Hospital Rehabilitation

**DOI:** 10.3390/medicina62071284

**Published:** 2026-07-03

**Authors:** By Arturo Olazabal, Sri Banerjee, Thomas O’Grady

**Affiliations:** 1 College of Health Sciences and Public Policy, Walden University, Minneapolis, MN 55401, USA; srikanta.banerjee2@mail.waldenu.edu (S.B.); thomas.ogrady@mail.waldenu.edu (T.O.); 2 Department of Population Health and Clinical Services, Lakeview Specialty Hospital and Rehabilitation, Waterford, WI 53185, USA

**Keywords:** traumatic brain injury, rehabilitation, long-term acute care hospital, LTACH, postacute care, Section GG, functional outcomes, mobility, activities of daily living, neurorehabilitation

## Abstract

*Background and Objectives*: Evidence guiding rehabilitation outcomes among medically complex traumatic brain injury (TBI) populations remains limited, particularly in long-term acute care hospital (LTACH) settings. Most prior studies have focused on inpatient rehabilitation facilities, leaving a limited understanding of factors influencing recovery during LTACH rehabilitation. This study examined the association between mechanism of injury, time from injury to LTACH admission, and functional rehabilitation outcomes in adults with moderate-to-severe TBI. *Materials and Methods*: This retrospective observational cohort study included adults with moderate-to-severe TBI who received multidisciplinary rehabilitation in an LTACH between January 2017 and December 2024. Of 272 eligible patients, 239 met the inclusion criteria after exclusions for short length of stay, incomplete evaluations, duplicate records, death during rehabilitation, or full independence at admission. Functional improvement in mobility and activities of daily living (ADLs) was measured using CMS Section GG assessments and dichotomized as improvement versus no improvement. Logistic regression analyses examined associations between predictors and outcomes while adjusting for demographic covariates. *Results*: Longer time from injury to LTACH admission was associated with lower odds of functional improvement. Compared with admission within 30 days, admission at 31–60 days (*OR* = 0.53; 95% CI, 0.28–0.99; *p* = 0.045) and beyond 60 days (*OR* = 0.26; 95% CI, 0.10–0.69; *p* = 0.006) was associated with reduced odds of improvement. Mechanism of injury was not significantly associated with outcomes. Male gender was associated with higher odds of improvement (*OR* = 2.18; 95% CI, 1.05–4.51; *p* = 0.036). No significant interaction effects were identified. *Conclusions*: Delayed LTACH admission was associated with lower odds of functional improvement following TBI rehabilitation, independent of injury mechanism and demographic factors. These findings support the importance of timely access to postacute rehabilitation among medically complex TBI populations.

## 1. Introduction

Traumatic brain injury (TBI) is a major public health concern associated with substantial long-term disability, healthcare utilization, and socioeconomic burden worldwide [1,2,3]. Recent estimates indicate that approximately 55 million individuals live with the long-term effects of TBI globally, with moderate-to-severe injuries frequently resulting in persistent impairments in mobility, activities of daily living (ADLs), cognition, and psychosocial functioning that require prolonged multidisciplinary rehabilitation [2,3,4,5]. Early and coordinated rehabilitation has been associated with improved functional recovery, shorter length of stay, and reduced disability following TBI [6,7,8]. However, rehabilitation outcomes vary considerably across postacute care settings and patient populations.

Long-term acute care hospitals (LTACHs) represent a specialized phase of postacute care for medically complex patients who are not yet appropriate candidates for traditional inpatient rehabilitation facilities [9,10]. Patients admitted to LTACHs often require prolonged medical management, ventilatory support, and specialized interdisciplinary rehabilitation following severe neurological injury [11,12]. Recent studies have demonstrated that patients with TBI admitted to LTACHs are typically older, medically complex, and at increased risk for prolonged functional impairment and mortality [9,11]. Despite the important role of LTACHs within the rehabilitation continuum, evidence examining functional outcomes and predictors of recovery in this setting remains limited [13]. Most prior rehabilitation studies have focused on inpatient rehabilitation facilities, skilled nursing facilities, or acute care populations [14,15,16]. As a result, rehabilitation trajectories during the LTACH phase remain incompletely understood.

Mechanism of injury and timing of rehabilitation initiation are recognized as important factors influencing recovery after TBI [2,6,8]. Earlier rehabilitation has consistently been associated with improved functional outcomes, whereas delayed rehabilitation may contribute to poorer recovery trajectories and prolonged disability [7,8,17]. For example, Bae and Lee [17] reported substantially longer lengths of stay among patients with delayed rehabilitation initiation following TBI. However, evidence regarding these associations during LTACH rehabilitation remains scarce [13]. Previous studies have also reported inconsistent findings regarding the influence of demographic characteristics, including age, gender, and race/ethnicity, on rehabilitation outcomes following TBI [2,18,19]. Clarifying these relationships may support more targeted rehabilitation planning in medically complex populations.

Guided by the Social Ecological Model, recovery was conceptualized as the result of interacting individual, interpersonal, organizational, and health-system factors [20,21,22]. The Social Ecological Model has also been widely applied to complex public health and rehabilitation contexts requiring coordinated systems-level approaches [23]. Our study addressed the limited evidence examining predictors of functional rehabilitation outcomes during the LTACH phase of recovery after TBI. Little is known regarding the influence of injury mechanism and rehabilitation timing on functional outcomes among medically complex patients receiving LTACH rehabilitation. We examined the association between mechanism of injury, time from injury to LTACH admission, and functional rehabilitation outcomes among adults with moderate-to-severe TBI receiving postacute rehabilitation in an LTACH setting. It was hypothesized that a longer time from injury to rehabilitation would be associated with lower odds of functional improvement. The findings demonstrated that delayed admission to LTACH rehabilitation was independently associated with reduced odds of functional improvement, whereas mechanism of injury was not significantly associated with outcomes.

## 2. Materials and Methods

### 2.1. Study Design and Setting

This study employed a retrospective cohort design using secondary data obtained from a long-term acute care hospital (LTACH) system in the Midwestern United States to examine the association between mechanism of injury, time from injury to rehabilitation initiation, and functional rehabilitation outcomes among adults with TBI receiving postacute rehabilitation in a LTACH. The study was conducted using retrospective clinical rehabilitation data collected between January 2017 and December 2024.

### 2.2. Participants

Participants included adults aged ≥18 years with moderate-to-severe TBI who received multidisciplinary rehabilitation targeting mobility and activities of ADLs during LTACH hospitalization. Inclusion criteria required completion of both admission and discharge functional assessments. Exclusion criteria included length of stay < 7 days, incomplete rehabilitation evaluations, duplicate records, death during rehabilitation, or full functional independence at admission.

A total of 272 patients met initial eligibility criteria. After exclusions, 239 participants were included in the final analysis.

### 2.3. Variables and Outcomes Measures

The primary independent variables were mechanism of injury and time from injury to LTACH admission. Mechanism of injury was categorized as motor vehicle accidents, falls, or violence-related injuries. Time from injury was categorized as ≤30 days, 31–60 days, or >60 days. Covariates included age, gender, and race/ethnicity.

The primary outcome was improvement in functional rehabilitation, as measured by CMS Section GG standardized functional assessments [24,25] of mobility and ADLs. Functional improvement was assessed during the LTACH rehabilitation episode, with outcomes measured from admission to discharge, using CMS Section GG assessments completed as part of routine clinical care. The CMS Section GG functional performance coding scale is presented in Appendix A. Occupational and physical therapists completed Section GG assessments at admission and discharge as part of routine clinical care. Composite functional scores were calculated by combining mobility and ADL performance measures. Functional progress was calculated as the Section GG differential score (final − goal), reflecting each patient’s discharge performance relative to individualized therapy goals established at admission. Although rehabilitation outcomes are frequently evaluated using admission-to-discharge change scores, goal-attainment approaches have also been used in rehabilitation research to assess recovery relative to individualized functional targets. Similar goal-oriented approaches have been described in postacute rehabilitation research, and the present methodology is conceptually consistent with CMS functional outcome frameworks that incorporate expected and discharge functional performance [14,25].

### 2.4. Statistical Analysis

Descriptive statistics summarize demographic and clinical characteristics. Chi-square tests and Fisher–Freeman–Halton exact tests were used to examine unadjusted associations between categorical variables and functional outcomes. Binary logistic regression analyses were performed to estimate associations between predictors and functional improvement while adjusting for demographic covariates. Odds ratios (*OR*s) and 95% confidence intervals (CIs) were reported. Interaction analyses examined whether demographic variables modified the association between time from injury and rehabilitation outcomes. Statistical significance was defined as *p* < 0.05. Analyses were conducted using SPSS version 30.0 (IBM Corp., Armonk, NY, USA).

### 2.5 Use of Artificial Intelligence

During the preparation of this work, the authors used ChatGPT (Model GPT-4o) for language correction. After using these tools/services, the authors reviewed and edited the content as needed and therefore take full responsibility for the content of the publication.

## 3. Results

### 3.1. Participant Characteristics

A total of 272 patients with TBI were screened for eligibility between January 2017 and December 2024. After applying predefined exclusion criteria, 239 participants were included in the final analysis. The most common reasons for exclusion were length of stay < 7 days and incomplete rehabilitation evaluations. Motor vehicle accidents were the most common mechanism of injury (56.5%), followed by falls (36.4%) and violence-related injuries (7.1%) (Table 1). Functional improvement in mobility and/or activities of daily living (ADLs) was observed in 33.1% of participants (Table 1).

### 3.2. Regression Analysis

Bivariate analyses demonstrated a significant association between time from injury to LTACH admission and functional improvement status, χ^2^(2, N = 239) = 9.34, *p* = 0.009, whereas mechanism of injury was not significantly associated with rehabilitation outcomes, χ^2^(2, N = 239) = 2.85, *p* = 0.241. Similarly, no significant unadjusted associations were observed between functional improvement and gender or race/ethnicity. Unadjusted logistic regression analyses (Table 2) demonstrated a nonsignificant trend toward lower odds of functional improvement among participants admitted 31–60 days after injury compared with those admitted within 30 days *(OR* = 0.56; 95% CI, 0.31–1.03; *p* = 0.062). Admission beyond 60 days was associated with significantly lower odds of functional improvement (*OR* = 0.33; 95% CI, 0.13–0.82; *p* = 0.017).

After adjustment for age, gender, and race/ethnicity (Table 3), admission at 31–60 days was associated with significantly lower odds of functional improvement compared with admission within 30 days (*OR* = 0.53; 95% CI, 0.28–0.99; *p* = 0.045). Admission beyond 60 days demonstrated an even greater reduction in the odds of improvement (*OR* = 0.26; 95% CI, 0.10–0.69; *p* = 0.006). Mechanism of injury was not significantly associated with functional outcomes. Male gender was independently associated with higher odds of functional improvement (*OR* = 2.18; 95% CI, 1.05–4.51; *p* = 0.036), whereas age and race/ethnicity were not significant predictors.

### 3.3. Interaction Analysis

Interaction analyses evaluated whether demographic variables modified the association between time from injury and functional rehabilitation outcomes. No statistically significant interaction effects were identified between time from injury and age, gender, or race/ethnicity (Table 3). These findings indicate that the association between delayed rehabilitation and lower functional improvement was generally consistent across demographic subgroups.

## 4. Discussion

To our knowledge, this study is among the first to examine the association between mechanism of injury, time from injury to LTACH admission, and functional rehabilitation outcomes among adults with moderate-to-severe TBI receiving postacute rehabilitation in an LTACH setting. The findings demonstrated that delayed admission to LTACH rehabilitation was independently associated with lower odds of functional improvement in mobility and ADLs. In contrast, mechanism of injury was not significantly associated with rehabilitation outcomes. These findings support the study hypothesis that longer delays between injury and rehabilitation initiation are associated with poorer functional recovery. However, because detailed measures of injury severity were unavailable, the possibility that delayed admission served as a proxy for greater initial neurological injury cannot be excluded.

Time from injury emerged as the strongest predictor of rehabilitation outcomes in this cohort. Participants admitted more than 30 days after injury demonstrated significantly lower odds of improvement, with the greatest reduction observed among individuals admitted after 60 days. These findings are consistent with previous rehabilitation literature demonstrating that earlier rehabilitation initiation is associated with improved functional outcomes, shorter length of stay, and reduced disability following TBI [6,7,8,17]. Prior studies conducted in inpatient rehabilitation and acute care settings have similarly reported improved mobility and ADL outcomes among patients receiving earlier rehabilitation interventions [14,15,16]. Collectively, these findings reinforce the importance of timely access to postacute rehabilitation services during critical recovery phases after TBI.

The mechanism of injury was not significantly associated with functional outcomes in this study. Although motor vehicle accidents were the most common injury mechanism, rehabilitation outcomes did not differ significantly across injury categories. Previous studies have reported associations between injury mechanism, injury severity, and acute neurological outcomes [2,19,26]. However, the present findings suggest that within medically complex LTACH populations, rehabilitation timing and postacute clinical factors may have a greater influence on functional recovery than the initial cause of injury. This may reflect the shared medical complexity and prolonged recovery trajectories characteristic of LTACH patients.

The overall rate of functional improvement observed in this cohort was lower than rates commonly reported in inpatient rehabilitation populations [14,15,16]. This finding likely reflects the substantial medical complexity of LTACH patients, who frequently require prolonged medical management, ventilatory support, and other medical complexities that delay rehabilitation initiation [9,10,11,12,13]. Nevertheless, approximately one-third of participants achieved measurable functional improvement, supporting the clinical value of multidisciplinary rehabilitation in medically complex TBI populations.

Demographic variables did not significantly modify the relationship between time from injury and rehabilitation outcomes. These findings suggest that delayed rehabilitation may adversely affect recovery across demographic subgroups. Previous studies have reported inconsistent associations between demographic factors and TBI outcomes [2,18,19]. The present findings emphasize the broader importance of reducing delays in access to postacute rehabilitation services.

From a systems perspective, delayed admission to LTACH rehabilitation may reflect barriers related to referral processes, insurance authorization, medical instability, or limited availability of specialized postacute services. The findings, therefore, highlight the importance of coordinated care transitions and streamlined referral pathways to facilitate timely access to LTACH rehabilitation for medically complex patients with TBI.

### 4.1. Limitations

Several limitations should be considered. The retrospective cohort design precludes causal inference, and the study was conducted in a single LTACH, which may limit generalizability. Important measures of TBI severity, including Glasgow Coma Scale scores, duration of post-traumatic amnesia, neuroimaging findings, neurosurgical interventions, and mechanical ventilation requirements, were not available within the dataset. Consequently, the observed association between delayed LTACH admission and lower odds of functional improvement may partially reflect differences in initial injury severity that could not be fully accounted for in the analysis. Rehabilitation intensity and certain social determinants of health were also unavailable. Patients who died during rehabilitation, had a length of stay less than seven days, had incomplete functional assessments, or were functionally independent at admission were excluded from the analysis. Although these exclusions were necessary to ensure meaningful evaluation of rehabilitation outcomes, they may limit generalizability to the broader population of patients with TBI receiving LTACH care, particularly those with extremely severe illness, abbreviated rehabilitation stays, or minimal functional impairment at admission. Functional outcomes were dichotomized, potentially reducing statistical power and sensitivity to smaller, yet clinically meaningful, differences in functional performance. Although this approach facilitated the interpretation of the likelihood of achieving functional improvement, some information in the original functional scores may have been lost. Rehabilitation outcomes were defined using a goal-attainment approach rather than the more commonly reported admission-to-discharge change score. Although this methodology reflects individualized rehabilitation expectations, it may limit direct comparison with studies using traditional functional change measures and may be influenced by variability in therapist goal-setting practices. Additionally, functional outcomes were assessed during the LTACH rehabilitation episode from admission to discharge rather than at a standardized follow-up interval. Because functional status immediately following injury was unavailable, the potential influence of spontaneous neurological recovery occurring before LTACH admission could not be fully evaluated.

### 4.2. Recommendations

Future research should examine rehabilitation timing and functional recovery using longitudinal and multicenter designs. Studies incorporating standardized measures of injury severity, including Glasgow Coma Scale scores, post-traumatic amnesia, neuroimaging findings, neurosurgical interventions, rehabilitation intensity, medical complexity, and social determinants of health may further clarify factors influencing postacute TBI recovery in LTACH settings. Additionally, the use of continuous functional outcome measures, standardized follow-up intervals, and rehabilitation length of stay as potential predictors may improve understanding of recovery trajectories following moderate-to-severe TBI.

## 5. Conclusions

Delayed admission to LTACH rehabilitation following TBI was associated with lower odds of functional improvement in mobility and activities of daily living, independent of mechanism of injury and demographic characteristics. In contrast, the mechanism of injury was not significantly associated with rehabilitation outcomes in this medically complex population. These findings emphasize the importance of timely access to specialized postacute rehabilitation services following moderate-to-severe TBI. Within LTACH settings, system-level factors related to rehabilitation timing and care transitions may have greater influence on early functional recovery than injury etiology alone. Efforts to reduce delays in referrals and admission to postacute rehabilitation may help improve recovery trajectories among medically complex patients with TBI. Further multicenter and longitudinal studies are warranted to better characterize rehabilitation timing, medical complexity, and long-term functional outcomes across postacute care settings.

## Figures and Tables

**Table 1:** Characteristics of the study participants.

Characteristics	Full Sample *N* = 239 (100%)	Functional Differential Score		*p* Value
		No Improvement *N* = 160 (66.9%)	Improvement *N* = 79 (33.1%)	
Mechanism of injury				0.241
MVA	135 (56.5)	92 (68.1)	43 (31.9)	
Fall	87 (36.4)	54 (62.1)	33 (37.9)	
Violence	17 (7.1)	14 (82.4)	3 (17.6)	
Time from injury				0.009
≤30 days	111 (46.4)	64 (57.7)	47 (42.3)	
31–60 days	89 (37.2)	64 (71.9)	25 (28.1)	
>60 days	39 (16.3)	32 (82.1)	7 (17.9)	
Age (y), Mean (SD)	48.93 (16.314)			
Gender				0.111
Female	54 (22.6)	41 (75.9)	13 (24.1)	
Male	185 (77.4)	119 (64.3)	66 (35.7)	
Race/ethnicity				0.637 ^1^
Black	34 (14.2)	23 (67.6)	11 (32.4)	
White	187 (78.2)	125 (66.8)	62 (33.2)	
Hispanic	8 (3.3)	4 (50.0)	4 (50.0)	
Other	10 (4.2)	8 (80.0)	2 (20.0)	

Note: Abbreviations: MVA = Motor vehicle accident. Values represent frequencies and percentages of participants with functional improvement by mechanism of injury and time from injury to rehabilitation initiation. Statistical significance was set at *p* < 0.05. Statistical significance was determined at *p* < 0.05. ^1^ Fisher’s Feeman-Halton exact tests were used when expected cell counts were <5.

**Table 2:** Unadjusted logistic regression analysis of mechanism of injury and time from injury on functional rehabilitation outcomes.

Predictor (Reference)	*OR*	95% CI	*p* Value
Mechanism of injury (ref.: MVA)			
Falls vs. MVA	1.21	[0.68, 2.16]	0.513
Violence vs. MVA	0.63	[0.17, 2.38]	0.493
Time from injury (ref.: ≤30 days)			
31–60 days vs. ≤30 days	0.56	[0.31, 1.03]	0.062
>60 days vs. ≤30 days	0.33	[0.13, 0.82]	0.017

Note. Abbreviations: MVA = Motor vehicle accident. Outcome modeled as a binary rehabilitation outcome. Reference categories: mechanism of injury = motor vehicle accident; time from injury = ≤30 days.

**Table 3:** Adjusted multivariable logistic regression analysis of mechanism of injury, time from injury, and demographic covariates on functional rehabilitation outcomes.

Predictor (Reference)	*OR*	95% CI	*p* Value
Mechanism of injury (ref.: MVA)			
Fall vs. MVA	1.62	[0.82, 3.17]	0.163
Violence vs. MVA	0.58	[0.15, 2.29]	0.435
Time from injury (ref.: ≤30 days			
31–60 days vs. ≤30 days	0.53	[0.28, 0.99]	0.045
>60 days vs. ≤30 days	0.26	[0.10, 0.69]	0.006
Age (per year)	0.99	[0.97, 1.01]	0.241
Gender (male vs. female)	2.18	[1.05, 4.51]	0.036
Race/ethnicity (ref.: Black)			
White vs. Black	0.68	[0.28, 1.65]	0.398
Hispanic vs. Black	1.86	[0.36, 9.60]	0.461
Other vs. Black	0.39	[0.07, 2.30]	0.296

Note. Abbreviations: MVA = Motor vehicle accident. Outcome modeled as a binary rehabilitation outcome. Reference categories: mechanism of injury = motor vehicle accident; time from injury = ≤30 days.

## Data Availability

The data presented in this study are available on request from the corresponding author due to institutional and privacy restrictions involving de-identified clinical rehabilitation data. Data may be available from the corresponding author upon reasonable request and with appropriate institutional approval.

## Abbreviations

The following abbreviations are used in this manuscript:

ADLs	Activities of Daily Living
CDC	Centers for Disease Control and Prevention
CI	Confidence Interval
CMS	Centers for Medicare & Medicaid Services
HIPAA	Health Insurance Portability and Accountability Act
IMPACT Act	Improving Medicare Post-Acute Care Transformation Act
LTACH	Long-Term Acute Care Hospital
NASEM	National Academies of Sciences, Engineering, and Medicine
OR	Odds Ratio
SPSS	Statistical Package for the Social Sciences
TBI	Traumatic Brain Injury

## Appendix A

### Appendix A.1. Functional Performance Coding for ADLs and Mobility (Section GG)

CMS Section GG coding scale used to assess functional performance in Activities of Daily Living (ADLs) and Mobility. Each item is rated from 0 to 6, reflecting the level of assistance required.

**Table A1:** Coding Scale.

Code	Explanation
0	Activity Not Attempted—not performed due to safety or medical condition.
1	Dependent—complete assistance required.
2	Substantial/Maximal Assistance—patient performs < 50% of effort.
3	Partial/Moderate Assistance—patient performs ≥ 50% of effort.
4	Supervision or Touching Assistance—needs supervision or light contact.
5	Setup or Clean-Up Assistance—needs only preparation or clean-up help.
6	Independent—no assistance needed.

Per CMS directive, “activity not attempted” reasons (07 = patient refused; 09 = not applicable (patient did not perform the task before stroke); 10 = not attempted because of environmental limitations; 88 = not attempted because of medical condition or safety concerns) were scored “1”.

### Appendix A.2. ADLs (Evaluated by Occupational Therapy)

(a) Eating
(b) Oral hygiene
(c) Toileting hygiene
(d) Showering/bathing self
(e) Washing lower body
(f) Upper body dressing
(g) Lower body dressing
(h) Toilet transfer (on/off)
(i) Bed/chair transfer

### Appendix A.3. Mobility (Evaluated by Physical Therapy)

(a) Bed mobility (roll left/right/back)
(b) Sit to lie
(c) Lying to sitting
(d) Sit to stand
(e) Bed-to-chair/chair transfer
(f) Toilet transfer (on/off)
(g) Walking 10 feet
(h) Walking 50 feet with two turns
(i) Walking 150 feet
(j) Four steps
(k) Wheel 50 feet with two turns
(l) Wheel 150 feet
